# Parallel Manipulation and Flexible Assembly of Micro-Spiral *via* Optoelectronic Tweezers

**DOI:** 10.3389/fbioe.2022.868821

**Published:** 2022-03-21

**Authors:** Shuzhang Liang, Jiayu Sun, Chaonan Zhang, Zixi Zhu, Yuguo Dai, Chunyuan Gan, Jun Cai, Huawei Chen, Lin Feng

**Affiliations:** ^1^ School of Mechanical Engineering and Automation, Beihang University, Beijing, China; ^2^ Beijing Advanced Innovation Center for Biomedical Engineering, Beihang University, Beijing, China

**Keywords:** optoelectronic tweezers, micro-spiral, parallel manipulation, flexible microassembly, micro-/nanorobotics

## Abstract

Micro-spiral has a wide range of applications in smart materials, such as drug delivery, deformable materials, and micro-scale electronic devices by utilizing the manipulation of electric fields, magnetic fields, and flow fields. However, it is incredibly challenging to achieve a massively parallel manipulation of the micro-spiral to form a particular microstructure in these conventional methods. Here, a simple method is reported for assembling micro-spirals into various microstructures *via* optoelectronic tweezers (OETs), which can accurately manipulate the micro-/bio-particles by projecting light patterns. The manipulation force of micro-spiral is analyzed and simulated first by the finite element simulation. When the micro-spiral lies at the bottom of the microfluidic chip, it can be translated or rotated toward the target position by applying control forces simultaneously at multiple locations on the long axis of the micro-spiral. Through the OET manipulation, the length of the micro-spiral chain can reach 806.45 μm. Moreover, the different parallel manipulation modes are achieved by utilizing multiple light spots. The results show that the micro-*spirulina* can be manipulated by a real-time local light pattern and be flexibly assembled into design microstructures by OETs, such as a T-shape circuit, link lever, and micro-coil pairs of devices. This assembly method using OETs has promising potential in fabricating innovative materials and microdevices for practical engineering applications.

## Introduction

The spiral structure is typical, such as the DNA double helix (Hu, 2021). It has distinctive properties and advantages over the conventional spherical, rod, or lamellar particles (Dong et al., 2022). Therefore, the spiral structure had a wide range of applications in various fields in recent years (Berny et al., 2020; Chandler et al., 2020; Wu et al., 2021). For example, the spiral structure can be propelled by rotating itself around the long axis. Thus, it is an excellent propulsion method in low-Reynolds number fluids. Micro-spirals are used as magnetic microrobots to achieve targeted drug delivery (Yan et al., 2015; Yan et al., 2017). The propelled velocity of the micro-spiral in fluids by a rotating magnetic field (Tottori et al., 2012) can reach 320 μm/s. Second, the spiral structure itself can exhibit superelasticity like a spring (Li et al., 2017). A stretchable supercapacitor (Shang et al., 2015), fabricated by a carbon nanotube double helix winding structure, can work under 150% stretching conditions. A stretching cycle of a flexible strain sensor with a micro-spiral can reach 5000 cycles (Li et al., 2017). Finally, the spiral structure can also induce a magnetic field by passing current or coupling with electromagnetic waves (Kamata et al., 2011; Cai et al., 2012). An electromagnetic material is fabricated by mixing metallic copper micro-spiral with paraffin (Kamata et al., 2014). The chiral characteristics and high-frequency response properties of the material in the 0.5–3 THz band are demonstrated. In particular, the materials fabricated by array micro-spirals can substantially improve mechanical enhancement, functional anisotropy, and biological tissue engineering (Gansel et al., 2009; Esposito et al., 2015).

To fabricate the materials or devices mentioned previously, micro-spirals are generally manipulated or assembled by applying external fields, such as electric, magnetic, or flow fields. For example, the DNA double helix (He et al., 2011) was conducted by using gate modulation of the surface charge of nanopore walls in an electric field. The electric field could significantly reduce the translocation velocity of DNA at a rate of 55 μm/s per 1 mV/nm. Multiple bio-template metallic helices (Li et al., 2016) were arranged into conductive micro-coil wires by alternating electric fields. Electromagnetically responsive THz structures (Kamata et al., 2014) were prepared by using micro-helix-based metal micro-spiral. Arrayed micro-helix terahertz metamaterials (Li et al., 2019) were designed by magnetron control so that materials with vertical helices exhibited significant chirality, while materials with horizontal helices exhibited about 20% polarization transition. The preparation of large-area arrays of micro-helices (Li et al., 2019) is achieved using a combination of flow and electric fields, reaching ∼70% of the particles assembled into chain-like structures for an area of 100 mm × 20 mm. These methods are used either to manipulate a single particle or to achieve the movement or arrangement of multiple particles together. Although these fields can manipulate and array micro-spiral, it is challenging to achieve the parallel independent manipulation of many micro-spiral particles. Thus, it is difficult to assemble the micro-spiral into various structures in the same microfluidic chip. Significantly, the conventional electric field or fluid field requires the preparation of specific electrodes or structures in the chip, which is costly and fixed.

Optoelectronic tweezers, different from conventional non-contact micro-/nanomanipulation technology [e.g., magnetic control (Lin et al., 2016; Dai et al., 2021), ultrasonic manipulation (Zhang W. et al., 2021), dielectrophoresis (Collet et al., 2015), and optical tweezers (Cheah et al., 2014)], utilize visual patterns to form virtual optical electrodes in a photoelectric layer. Then, it can generate a non-uniform electric field to achieve parallel independent manipulation of particles (Chiou et al., 2005), and adjusting the visual patterns can flexibly manipulate a large number of micro-/nano-objects (Liang et al., 2020; Puerto et al., 2020; Chu et al., 2021), such as cells (Yang et al., 2010; Chu et al., 2020), microorganisms (Mishra et al., 2016), and gold nanoparticles (Jamshidi et al., 2009). In addition, OETs can generate a primarily driven force with a low light intensity compared to optical tweezers by using light-induced dielectrophoresis. For example, 15,000 light spots were used to trap microparticles on a 1.3-mm^2^ × 1.0-mm^2^ chip at a projected light power of only 10 nW/μm by the OET system (Chiou et al., 2005). It also achieves patterned graphene devices (Lim et al., 2018), fabricated microelectrode (Meng et al., 2017), and arrayed rod-shaped particles (Iris et al., 2018). The movement velocity and rotational angular speed of microrobots (Zhang et al., 2019) can reach 1.1 mm/s and 9.7 rad/s, respectively, in the OET system. The manipulation force of the microsphere can reach 4.2 nN (Zhang et al., 2016). By parallel independent manipulation of the OET system, the micro-gear structure was assembled (Zhang S. et al., 2021). However, there are only a few reports of DNA manipulation in the OET system (Lin et al., 2009). Moreover, it seems that the micro-spiral structure was seen as a whole body, and the mechanism of the spiral structure was not clearly explained in the reference. In our previous work (Liang et al., 2021a; Liang et al., 2021b), the manipulation of particles with different dimensional shapes was achieved by OETs, and the size range of a single particle is from 2 to 150 μm. We first utilize the OETs to manipulate such a large-size micro-spiral structure.

**FIGURE 1:**
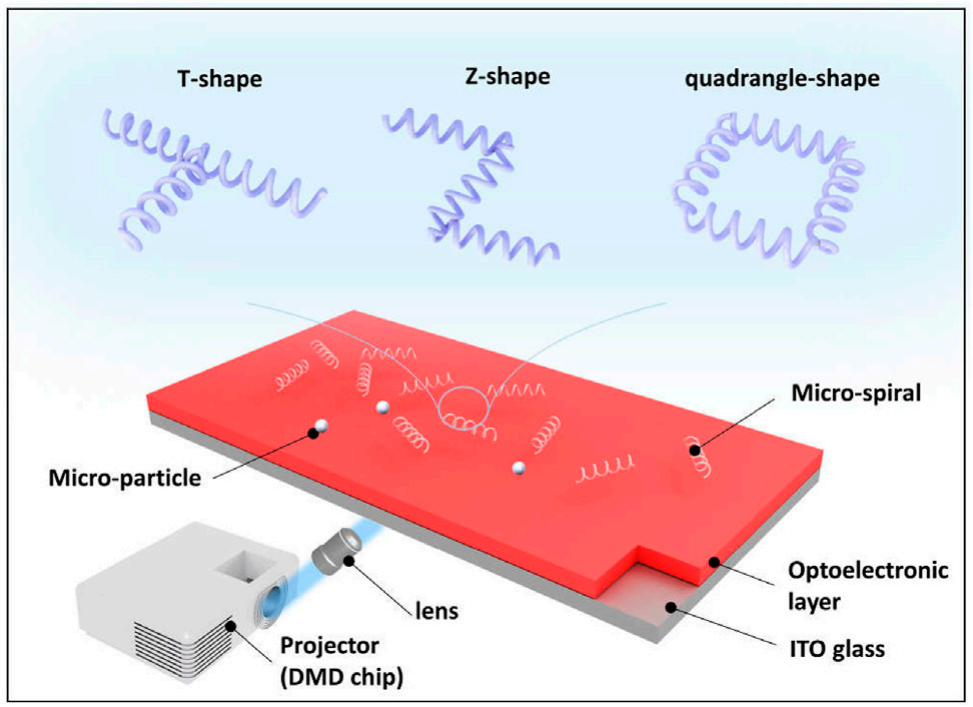
Conceptual overview of flexible assembly of micro-spirals *via* OETs.

Here, this study proposed massively parallel manipulation and flexible assembly of complex shape micro-spiral using optoelectronic tweezers, as shown in Figure 1. We first analyzed and simulated the micro-spiral dielectric properties for manipulation by the infinitesimal method. Based on these properties, the multiple light patterns are designed to achieve the joint manipulation of micro-*spirulina*. Subsequently, the micro-*spirulina* is parallely manipulated by different light spot modes. Finally, we applied the OETs to achieve a flexible assembly of micro-*spirulina* of different design microstructures: T-shaped circuits, Z-shaped linkages, and quadrangle-shaped micro-coil pairs. In the future, the present method is expected to be applied in the fabrication of innovative materials or microdevices.

## Materials and Methods

### Micro-Spiral Manipulation Theory

In the OET system, the main manipulation principle is based on the dielectrophoresis (DEP) theory (Hwang et al., 2008). When a dielectric particle is placed in the electric field, it will be polarized. Then, the particle will suffer a DEP force. If the electric field is non-uniform, the net force is not equal to zero. The particle will be attracted or repelled from the high-strength area of a non-uniform electric field. Generally, the spherical bioparticle is analyzed using the model called the multishell model, which can be can be equivalent to a single-shell model (Gagnon 2011; Jubery et al., 2014; Qian et al., 2014), as shown in Supplementary Figure S1. Here, we built an equivalent single-shell spiral model for demonstrating the moving characteristics. In addition, because the spiral can be divided into many cylinders segments (Dalir et al., 2009; Tao et al., 2015; Li et al., 2016), the depolarizing factor of three different axes is set to the same in any one cylinder segment for simplifying the calculation. Thus, the dielectric property parameters use the simplified sphere assumption model to analyze (For details, see Supplementary Theory). The direction force depended on the real part of the Clausius-Mossotti (CM) factor- Re[K(ω)] . It can be expressed as (Jubery et al., 2014; Qian et al., 2014):

$$Re\left[K\left(\omega \right)\right]=\frac{\omega^{2}\left(\varepsilon_{p}^{2}+\varepsilon_{p}\varepsilon_{m}-2\varepsilon_{m}^{2}\right)+\left(\sigma_{p}^{2}+\sigma_{p}\sigma_{m}-2\sigma_{m}^{2}\right)}{\omega^{2}{\left(\varepsilon_{p}+2\varepsilon_{m}\right)}^{2}+{\left(\sigma_{p}+2\sigma_{m}\right)}^{2}},$$ (1)

where *σ*_*p*_ and *ε*_*p*_ represent the electric conductivity and the permittivities of particles, respectively; *σ*_*m*_ and *ε*_*m*_ represent the electric conductivity and the permittivities of the medium, respectively; and *ω* is the angular frequency of the alternating (AC) source.

The real part of the CM factor mainly depends on the dielectric properties of particles, the suspending medium, and input frequency. If Re[K(ω)] is greater than zero, the particle will be subject to an attracted DEP force. Otherwise, it will be subject to a repelled DEP force. The direction of force is analyzed by calculation software (MATLAB 2021).

The sphere or rod particles generally use the equivalent dipole moment method to analyze the force (Tao et al., 2015). However, it is not easy to precisely calculate the force of the complex shape of micro-spiral particles. Here, the Maxwell stress tensor (MST) method (Rosales and Lim, 2005), a calculation model by integrating the stress tensor on the particle’s surface, is used to analyze the DEP force. The DEP force can be expressed as (Rosales and Lim, 2005):

$$F_{dep}=\oint \left(T\cdot n\right)\;dS=\oint \left(\left[Re\left(\varepsilon^{*}\right)\cdot \left(E\cdot E-\frac{1}{2}\left(E\cdot E\right)\cdot I\right)\right]\cdot n\right)\;dS,$$ (2)

where ***T*** represents the MST on the particle surface, ***n*** represents the unit vector normal to the particle surface, $\varepsilon^{*}=\varepsilon -\left(\frac{\sigma }{i\omega }\right)$ is the complex permittivity of the medium, ***E*** is the electric field, and ***I*** is the unit tensor. The finite element simulation software (COMSOL Multiphysics 5.5) is used to solve the input electric field and calculate the DEP force by using the MST formula (Eq. 2).

### a-Si:H-Based Microfluidic Chip and Optoelectronic Tweezer System

**FIGURE 2:**
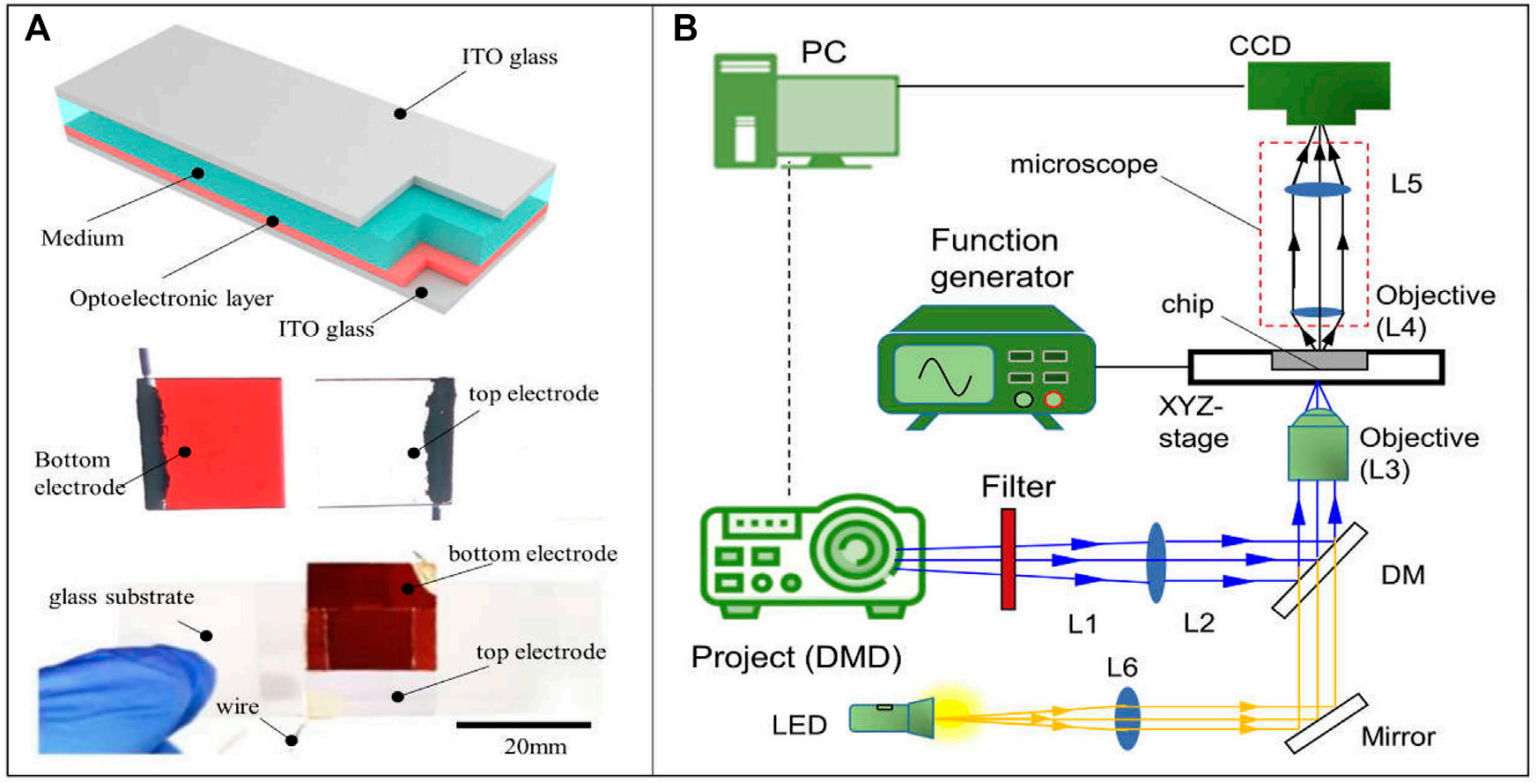
Simple schematic diagram of **(A)** the chip and **(B)** the OET.

In this study, the OET system utilizes the a-Si:H-based microfluidic chip to manipulate. The chip is shown in Figure 2A. It is a sandwich structure. The top electrode is indium tin oxide [ITO (NOZO Co., China)] glass (the size is 20 mm × 20 mm). The bottom electrode is ITO glass coated with a-Si:H film (the thickness is 1 μm and the size is 20 mm × 20 mm). The a-Si:H film is fabricated by a plasma-enhanced chemical vapor deposition (PECVD) system. These two electrodes are adhesive by a double-sided tape to form the meddle space (the thickness is 150 μm). The medium and sample can be injected into the area by a side inlet. The schematic of the OET system is shown in Figure 2B, and the image of the whole experiment system is given in Supplementary Figure S2. It consists of three parts. First, the image projected part is composed of a digital micromirror device (DMD) projector (Vivitek H1085, China), a filter, two reflecting mirrors (M), a lens, a dichroic mirror (DM), and an objective lens. The real-time light pattern can be projected onto the microfluidic chip for manipulation through the projected path. Second, the illuminated part comprises an LED, a focusing lens, a mirror (M), a dichroic mirror (DM), and an objective lens. The light generated from the LED can illuminate the microfluidic chip by the illuminated path. Third, the experimental observation part is a microscope (OLYMPUS SZX16, Japan), installed a charge-coupled device (CCD) camera (GS3-U3-23S6C-C; Canada). Through the CCD camera, the experimental processing can be observed in real time. In addition, an input electric field is generated by a function generator source. The light pattern is designed by using a computer. The computer is also used to receive practical information and achieve closed-loop control of the OET system.

### Preparation of the Micro-Spiral Sample

To demonstrate the manipulation of a micro-spiral using OETs, the *micro-spirulina*, a spiral-shaped aquatic plant, is selected as a sample. The size of *micro-spirulina* is listed as follows (Cai et al., 2019): length from 52 to 360 μm, helical thread diameter about 5–8 μm, helical thread diameter about 25–36 μm, and helical pitch about 43–57 μm. The *micro-spirulina* is filtered from the culture medium using a strainer. Then, 5 g *micro-spirulina* is put into the 50 ml deionized (DI) water as a manipulation sample medium. In addition, 10 μl of the *micro-spirulina* medium and 10 μl of 20-μm-diameter polystyrene microbeads (Zhongkeleiming; China) solution (50 mg/ml) are mixed in 1 ml of DI water. In the experiment of manipulation and assembly, 10 μl of *micro-spirulina* medium or the mixed solution is injected into the microfluidic chip of OETs using a pipette.

## Results and Discussion

### Simulation Parameter Analysis

**FIGURE 3:**
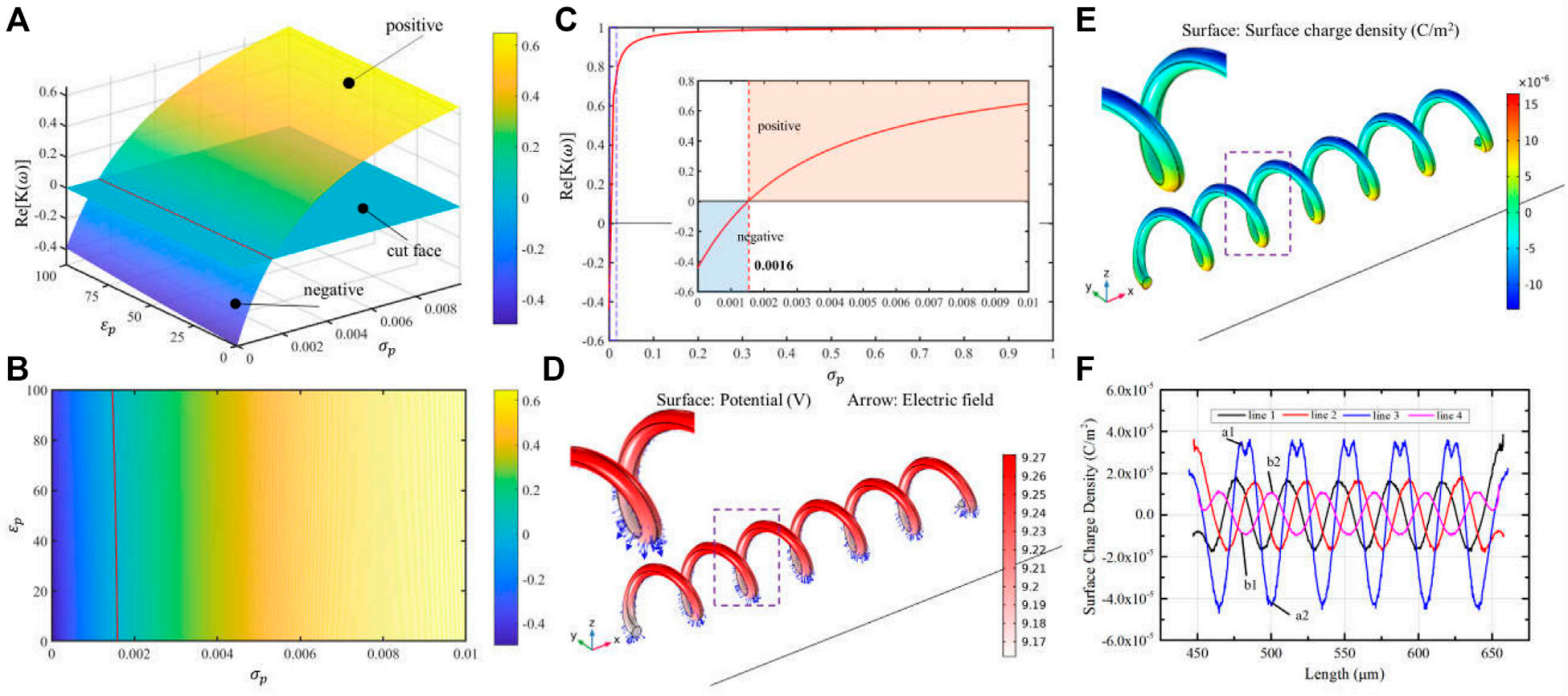
Simulation parameters of micro-*spirulina* analysis. **(A,B)** Relationship of the real part of CM factor-  Re[K(ω)] and permittivity and conductivity of micro-*spirulina.* Blue plane is a cut-face. Redline is an intersecting line between cut-face and Re[K(ω)] values surface. When the Re[K(ω)] values surface is over the cut-face, the *spirulina* will suffer an attracted force. Otherwise, it will suffer a repelled force. **(C)** Relationship of Re[K(ω)] and conductivity of micro-*spirulina*, when the permittivity of micro-*spirulina* is set as 58.52. **(D)** Electric potential and electric field distribution of micro-*spirulina* in an OET system. **(E,F)** Surface charge density of micro-*spirulina* when it was polarized in the OET.

As previously mentioned, the direction of manipulation force is first required to be considered in OETs, which decides the shape of the light pattern of manipulation. Equation 1 is used to analyze the direction of force according to medium and micro-object electric properties. However, there are a few reports about the electric properties of the original *micro-spirulina*. Here, the relationship between the direction of manipulation force and electric parameters of *micro-spirulina* is analyzed by MATLAB, as shown in Figures 3A–C. For the sample, the permittivity and conductivity of DI water are set at 80 and 1.5 × 10^−3^ S/m, respectively (Zhang et al., 2019). The frequency is set as 100 kHz, based on a previous experiment (Liang et al., 2021a). The results show that the value of permittivity and conductivity will be different. Furthermore, according to the permittivity of most biology mediums (Arai et al., 2010; Yang et al., 2010), we set the permittivity of micro-*spirulina* as 58.52 and then acquired the influence of conductivity to force direction, as shown in Figure 3C. With the mentioned condition, the threshold of direction is 0.0016 S/m. The conductivity of micro-*spirulina* is approximately measured as 0.04 S/m. Because this value is larger than the threshold value, the micro-*spirulina* suffers from a positive DEP force, which means it will be attracted by the optical pattern in the OET system.

There is some research about using MST methods to evaluate the DEP force and interaction force (Rosales and Lim, 2005; Ai and Qian, 2010). It is because the calculation accuracy of the MST method is higher than other methods for complex structure micro-objects (Rosales and Lim, 2005). In this study, the MST method is used to analyze the force of micro-spiral in the OET system. With these parameter conditions, we simulated the electric properties of micro-*spirulina* in OETs using the AC/DC and fluid–structure interaction module of COMSOL software, as shown in Figures 3D–F. The micro-*spirulina* usually lay on the bottom electrode, which presented the radius direction parallel to the electric field direction. Thus, the model is set as shown in Supplementary Figures S3A–C. The thickness of the a-Si:H layer is formed as 1 μm, and the dark conductivity and permittivity are set as 1 × 10^−6^ S/m and 11.7, respectively (Zhang et al., 2019). The thickness of the medium layer is formed as 150 μm. The sizes of micro-spiral are set as 30 μm in diameter, 6 μm in wire diameter, 35 μm in helical pitch, and 210 μm in the whole length. The distance between the micro-spiral and bottom electrode is 1 μm. The simulation results show that the side near the bottom electrode generates more surface charge than a further side. Four contour lines on the micro-spiral surface are selected to calculate the electric properties to analyze the polarized charge better, as shown in Supplementary Figure S3D. The micro-spiral accumulates more charge on the outside than inside. Because the electric field is uniform without a light pattern projected at this moment, the distribution of electric properties of micro-spiral is periodic along the axis direction, as shown in Figure 3F and Supplementary Figures S3E,F.

However, due to the micro-*spirulina* being closer to the bottom electrode, the strength of polarization of the bottom part of the micro-*spirulina* is larger than the top part (Pohl, 1978; Chu et al., 2021), as shown in Figure 3F. Supplementary Figure S3F shows the Maxwell stress tensor distribution. Thus, the net force is not equal to zero, and the micro-*spirulina* is attracted to the near electrode as a positive DEP effect. In addition, we also analyzed the polarization effect with the differently orientated micro-spiral models (the angle between the long axis of the spiral and bottom electrode are 45° and 90°, respectively), as shown in Supplementary Figure S4. When the micro-spiral is vertical, the polarized surface charge effect can be divided into the same segments because the electric field generates the same gradient difference in each segment. For other orientations, the polarized surface charge density forms a periodic vibration, which agrees with the spiral period.

### Demonstrate Multiple Light Patterns to Parallel Move and Rotate the Micro-Spiral

**FIGURE 4:**
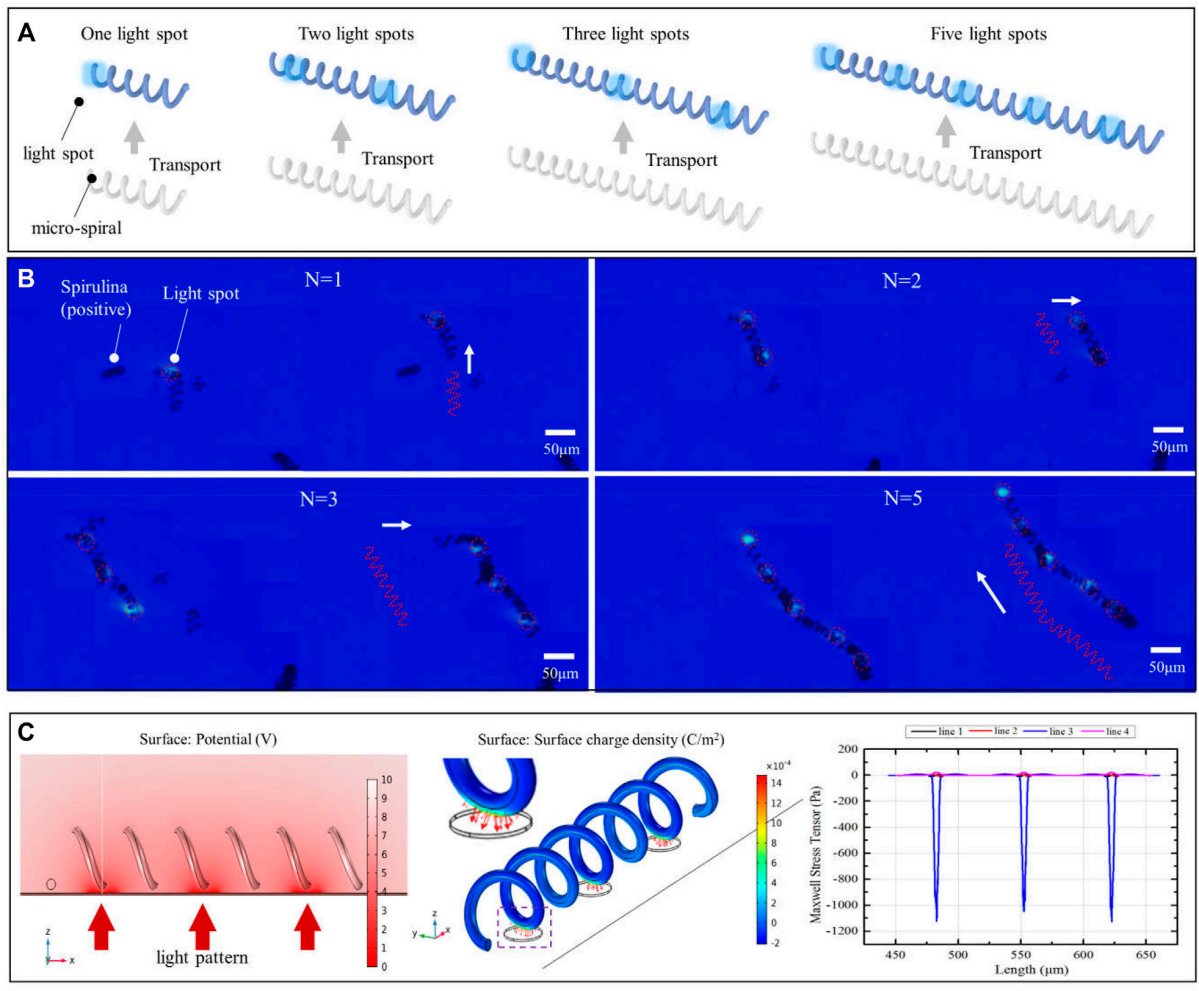
Multiple light spots to collaboratively transport the different length micro-*spirulina*. **(A)** Schematic of manipulation and **(B)** experimental results (the number of light spots is 1, 2, 3, and 5, respectively). **(C)** Simulations of electric potential, surface charge density, and Maxwell stress tensor.

**FIGURE 5:**
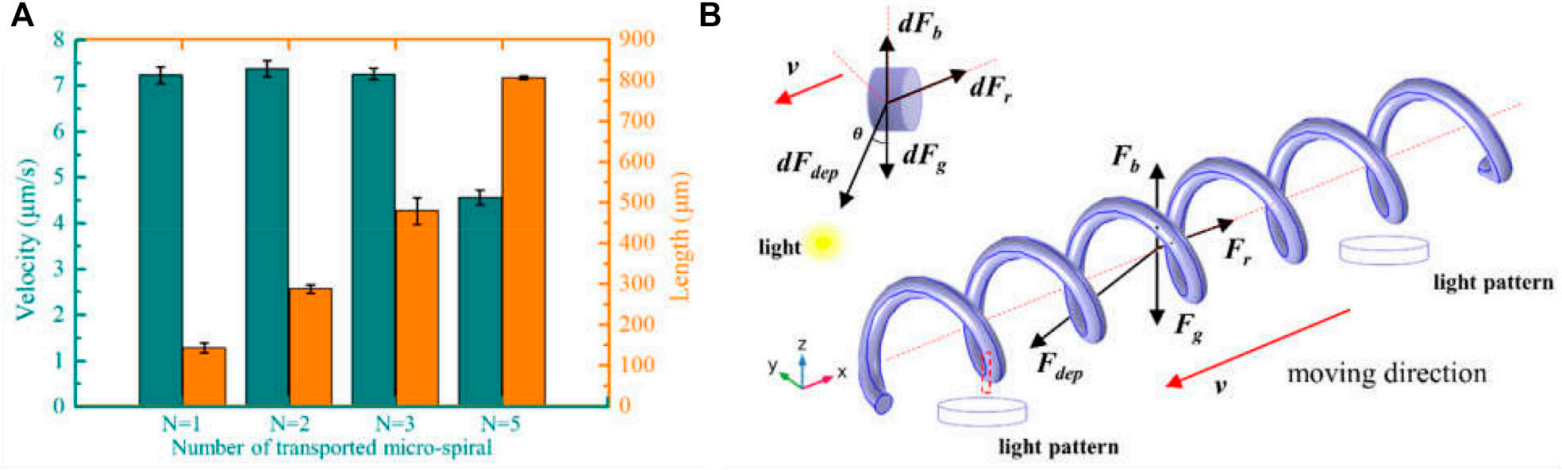
**(A)** Manipulation velocity of the different length of micro-*spirulina*. **(B)** An analysis of the manipulated force of micro-spiral in an OET.

Based on the positive DEP effect of micro-*spirulina* in the OET system, the light spots are designed to manipulate the complex shape. First, we utilized multiple light areas to collaboratively transport the different length micro-*spirulina* chains, which are made up of a different number of micro-*spirulina*, as shown in Figures 4A,B (a video clip is provided as a Supplementary Video S1). The input voltage and frequency are 10 V_pp_ and 100 kHz, respectively. The results show that the micro*-spirulina* was assembled into a chain, and the length can reach 806.45 μm. Moreover, the most length chain can be transported to the target position in the microfluidic chip with a velocity of 4.57 μm/s, as shown in Figure 5A. It also shows that the transportation velocity will decrease when the length is beyond a threshold even though the number of light spots increases simultaneously. This could be because the resistance forces have increased, and the gravity could not be ignored with the length of chains increase (Jubery et al., 2014). According to the Stokes law at low Reynolds number in OET system (Zhang 2016; Zhang et al., 2019), the manipulation force is calculated as 1.16pN (detail see Supplementary Theory). Furthermore, to probe the effect of manipulation of multiple light spots for the experimental results, simulations were also carried out in COMSOL Multiphysics. The simulation model is shown in Supplementary Figure S5A. Three light spots illuminate the optoelectric material, and the conductivity is 5 × 10^−3^ S/m. The diameter of light holes is 20 μm. The light spots induce a potential strength area and generate a non-uniform electric field, as shown in Figure 4C and Supplementary Figures S5B–D. Therefore, the micro-*spirulina* in these areas polarized more charge density, as shown in Supplementary Figure S5E. The electric field generates a positive force, as shown in Supplementary Figure S5F. An analysis of the manipulated force of micro-spiral in an OET is shown in Figure 5B. The micro-spiral is subject to dielectrophoresis force (*F*_*dep*_), resistance force (*F*_*r*_), gravity force (*F*_*g*_), and buoyant force (*F*_*b*_). These forces trapped the micro-*spirulina*—the simulation results agree with the manipulation effect in the OET system (see Supplementary Figure S6). When the light pattern moves, the direction of the force will change. The micro-spiral can move with the light pattern. This means utilizing multiple light spots to achieve cooperative manipulation of micro-spiral in a microfluidic chip compared with the three mentioned methods (electric, magnetic, or flow fields).

**FIGURE 6:**
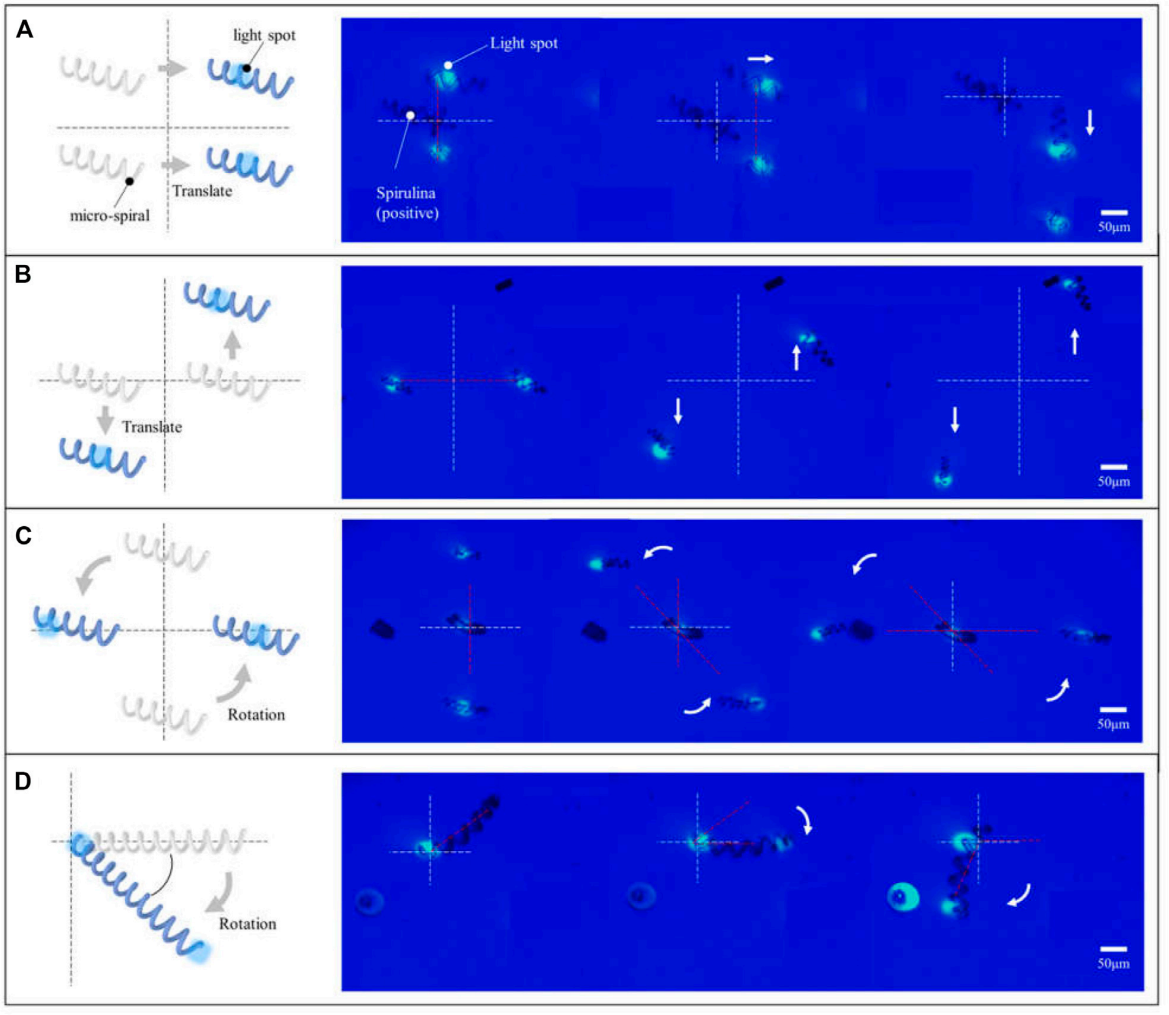
Micro-*spirulina* were parallely manipulated by multiple light spots in the OET system. Translated in the **(A)** same direction and **(B)** opposite direction. Rotating along with **(C)** a point in the bottom electrode and **(D)** a point of itself.

**FIGURE 7:**
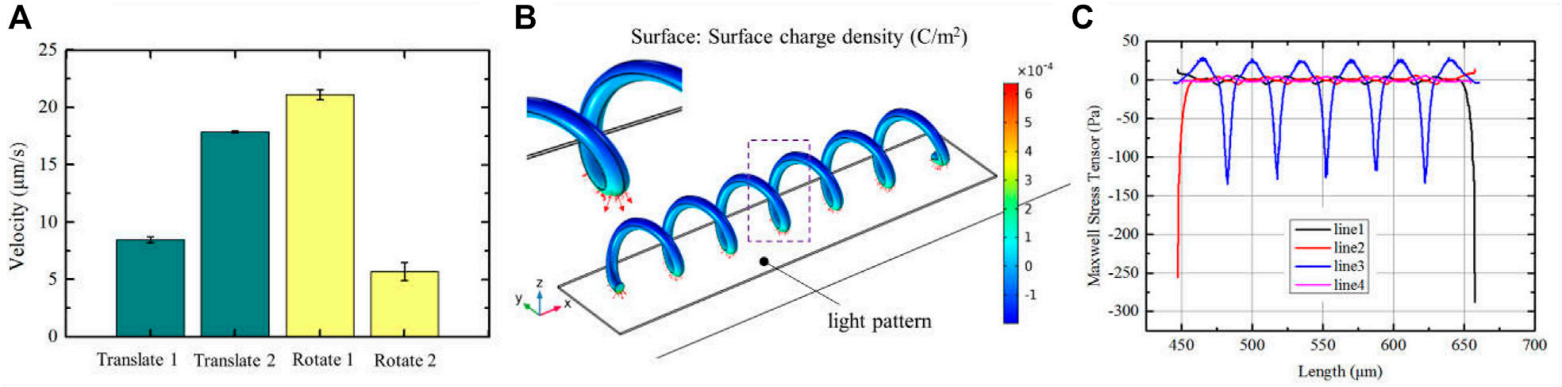
**(A)** Manipulation velocity of the different manipulation modes; simulation results of **(B)** surface charge density and **(C)** Maxwell stress tensor distribution when the light pattern with a little bit bigger than the length of micro-spiral.

In addition, the micro-*spirulina* was parallel manipulated by multiple light spots in the OET system. We designed the different manipulation modes. It shows that numerous micro-*spirulina* can be translated in the same direction or opposite direction, as shown in Figures 6A,B. The translation speed can reach 17.86 μm/s. In addition, two different rotation modes were carried out in the OET. One is two micro-*spirulina* rotating along with a point in the bottom electrode, as shown in Figure 6C. The other is the micro-*spirulina* spinning along with an end of itself, as shown in Figure 6D (a video clip is provided as a Supplementary Videos S2, S3). The linear speed of rotation can reach 21.13 μm/s. The manipulated force can reach 3.58 pN. As shown in Figure 7A, different manipulation modes may induce a sligthly influence on the manipulated velocity of micro-spiral. It mainly depends on particles, input source, and light pattern (Jubery et al., 2014; Liang et al., 2020) in the OET system. For the rotation along with a point of the micro-spiral, the velocity is smaller than other ways due to the cross-sectional area in the moving direction being larger than different ways. These manipulation modes indicate that the complex shape of micro-spiral could be flexibly manipulated using multiple light patterns in OETs.

On the other hand, we also analyzed the electric effect when the light spot is larger than the length of the structure (Zhang et al., 2022) (see Supplement Figure S7). The results of polarized surface charge and Maxwell stress tensor are shown in Figures 7B,C, respectively. In this condition, the polarized surface charge density forms a periodic variation, which agrees with the spiral period. The micro-spiral is confined within the optical pattern and moves with the light pattern together. These could affect the flexibility of manipulation. But, it can be applied to improve the stability in the transportation process.

### Flexibly Assemble Micro-Spiral Into Different Microstructures

**FIGURE 8:**
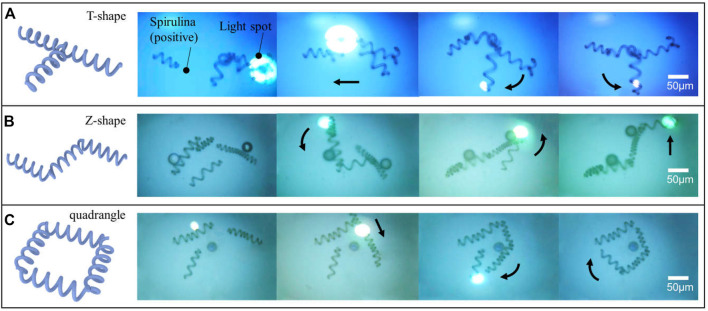
Flexible assembling micro-spiral into the different shapes of microstructure: **(A)** T-shape, **(B)** Z-shape, and **(C)** quadrangle shape.

To test the convenient control complex shape of micro-spiral with the aforementioned manipulation effect, we applied the OETs to assemble the micro-*spirulina*. As shown in Figure 8, the micro-spiral can be flexibly made as different shapes such as T-shape, Z-shape, and quadrangle shape (a video clip is provided as Supplementary Videos S4). Even though polystyrene particles adhered to the surface of micro-*spirulina* (Jones 1986; Liang et al., 2021a), they could also achieve assembly. This means that the assembly of micro-spiral could treat with special structure requirements and corporately assemble with other shape micro-objects (Zhang S. et al., 2021). Compared with the traditional assemble method (Fragouli et al., 2014; Li et al., 2016), this is easy to manipulate the micro-*spirulina* to form more specific shapes in one application device. Furthermore, utilizing the capacity of selecting OETs (Zhang et al., 2018; Puerto et al., 2020), the specific size design of assembly of micro-*spirulina* could sort from more samples improving the efficiency. In the future, we propose that the flexible assembly of the complex shape of micro-spiral by the OET system may be appropriate for assembling the complex vessel model structure. In addition, the automotive microassembly is also promising by programming a light pattern moving in the OET system (Liang et al., 2021b; Cao et al., 2021). We are confident that the reported method coupling with other methods would have many applications.

## Conclusion

This study aimed to achieve a flexible assembly of the complex shape of micro-spiral using the OET system. First, the direction of the manipulation force was analyzed. By using condition, the threshold value was 0.0016 S/m. Combined with the simulation result, the micro-*spirulina* was attracted by the optical pattern in the OET system. Utilizing the positive DEP effect, multiple light spots were applied to manipulate the micro-*spirulina* in a microfluidic chip of OET cooperatively. The results show that the micro-*spirulina* was linked to a chain with different lengths, and the size of the micro-spiral chain can reach 806.45 μm. Moreover, the chain can be transported to the target position by simultaneous working of multiple light spots. The length of the chain will influence the velocity of transportation because the resistance force and gravity will not be ignored beyond a threshold. Subsequently, the different manipulation modes were demonstrated utilizing the convenient controlled movement of the light spots. It shows that the micro-*spirulina* were parallely translated by multiple light spots in the same direction or opposite direction and rotated along with a point in the bottom electrode or a point of itself. The manipulation modes could generate a slight influence on speed. Finally, we applied the OET to flexibly assemble the micro-*spirulina* into design shapes of a T-shape circuit, link lever, and micro-coil pairs of devices. Furthermore, the assembly of micro-spiral could treat with special structure requirements and corporately assemble with other shape micro-objects. Based on this study, it is expected that the assembly method using the simple OET platform can be employed in intelligent materials medical and microdevice applications involving soft-sensor, micro-circuit, and micro-spring.

## Data Availability

The original contributions presented in the study are included in the article/Supplementary Material, further inquiries can be directed to the corresponding authors.
