# Supplementary material for: Parallel Manipulation and Flexible Assembly of Micro-Spiral via Optoelectronic Tweezers
Source: Front Bioeng Biotechnol. 2022 Mar 21;10:868821. doi: 10.3389/fbioe.2022.868821 (PMC8977588; doi:10.3389/fbioe.2022.868821)
Supplement: Supplementary file 2 [file DataSheet1.pdf]

Supplementary Material

1 Supplementary Theory

Generally, the spherical bioparticle is analyzed using the model called the multishell model (Jubery et al., 2014; Gagnon 2011; Qian et al., 2014), as shown in **Supplement Figure 1A**. Then, the multishell model can be equivalent to the double-shell model (as shown in **Supplement Figure 1B**) by a series of calculations. Finally, the double model also can be equivalent to a single-shell model (as shown in **Supplement Figure 1C**). Using the simplified single-shell sphere model, the effective complex permittivities(ε_p) and conductivity of bioparticles(σ_p) are expressed as (Jubery et al., 2014; Qian et al., 2014):

$$\varepsilon_p = \varepsilon_1 \frac{(\frac{R_1}{R_2})^2 + 2(\frac{\varepsilon_2 - \varepsilon_1}{\varepsilon_2 + 2\varepsilon_1})}{(\frac{R_1}{R_2})^2 - (\frac{\varepsilon_2 - \varepsilon_1}{\varepsilon_2 + 2\varepsilon_1})} \quad (1)$$

$$\sigma_p = \sigma_1 \frac{(\frac{R_1}{R_2})^3 + 2(\frac{\sigma_2 - \sigma_1}{\sigma_2 + 2\sigma_1})}{(\frac{R_1}{R_2})^3 - (\frac{\sigma_2 - \sigma_1}{\sigma_2 + 2\sigma_1})} \quad (2)$$

where, ε_1 and ε_2 are the complex permittivity of the bioparticle membrane and cytoplasm, respectively. R_1 and R_2 are the radius of the outermost layer and inner layer of bioparticles, respectively. σ_1 and σ_2 are the complex permittivity of the bioparticle membrane and cytoplasm, respectively.

Here, because cell walls are permeable, we ignore the influence of the dielectric properties of cell walls in this model. We try to build an ideal equivalent single-shell spiral model for demonstrated moving characteristics, and **eq. 1** and **2** are used to calculate the effective dielectric parameters in this simplified model. In addition, the spiral can be thought of as a lot of cylinders segments. According to the literature (Li et al., 2016; Tao et al., 2015; Dalir et al., 2009), the effective dielectric parameters could be used to analyze the property of the cylindrical shape. Thus, the CM factor is calculated by the effective complex permittivities and conductivity of the spiral. The preliminary simulation results show that the spiral is attracted by the light spot which agrees with the moving direction in the experiment. When the particle is a sphere, the dielectrophoresis force can be expressed as(Jubery et al., 2014; Gagnon 2011; Qian et al., 2014):

$$K(\omega) = \frac{\varepsilon_p^* - \varepsilon_m^*}{\varepsilon_p^* + 2\varepsilon_m^*} \quad (3)$$

Here, the spiral can be divided into a lot of cylinder segments. In order to simplify the calculation parameters, the depolarizing factor of three different axes is set to the same in any one cylinder segment. Therefore, the final model for calculation is approximately assumpted as the sphere.

In the OET system, the manipulation force can be calculated by Stokes's drag force(Zhang et al., 2019; Liang et al., 2020) which can be applied to measure the actual strength of DEP forces by the velocity of particles because the medium is laminar. Stokes's drag force is expressed as:

$$F_d = \frac{6\eta A u}{r} \quad (4)$$

Here, in the moving direction, the spiral suffers a resistance force with a ring cross-section (the outer diameter and inner diameters are R and r respectively), as shown in Figure 5. The feature size of spirulina is 30μm in diameter (D), 6μm in wire diameter (d). Thus, R=D/2+d/2=18μm, r=R-d=12μm. The resistance area of the ring is calculated to $\mathrm{A} = \pi(R^2 - r^2) \approx 3.14 \times (18^2 - 12^2) \times 10^{-12}\ m^2 = 5.652 \times 10^{-10}\ m^2$. Then, we used the resistance area to calculate an equivalent circle diameter r_e ($\mathrm{A} = \pi r_e^2$) for simplifying the calculation. r_e=1.342× 10^{-5}m. The spirulina was moved at a maximum linear velocity of 4.57 μm/s. The kinematic viscosity of the medium, deionized water, is η= 1 g/cm3. Therefore, according to Stokes's equation,

$$F_d = \frac{6\eta A u}{r_e} \approx \frac{6\times1.0\times10^{-3}\times5.652\times10^{-10}\times4.57\times10^{-6}}{1.342\times10^{-5}} = 1.155 \times 10^{-12} N \quad (5)$$

The spirulina suffers a drag force of approximately 1.155pN.

2 Supplementary Figures

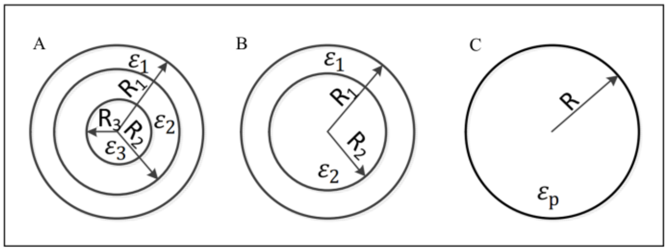

Supplementary Figure 1. Model of dielectric spherical bioparticles. (A) Multishell model; (B) Double-shell model; (C) Single-shell model.

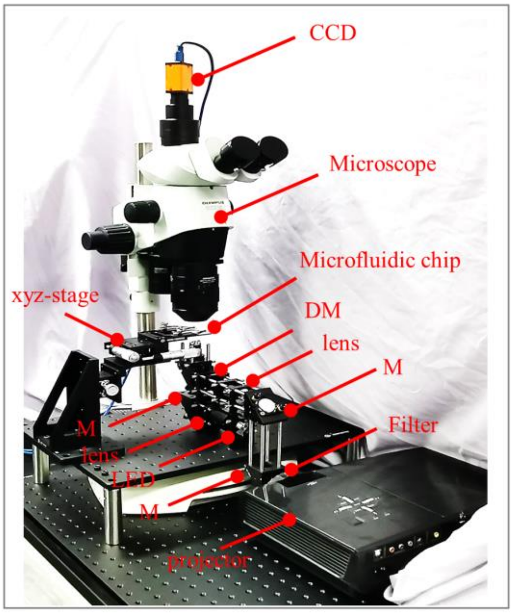

Supplementary Figure 2. The image of the OET system.

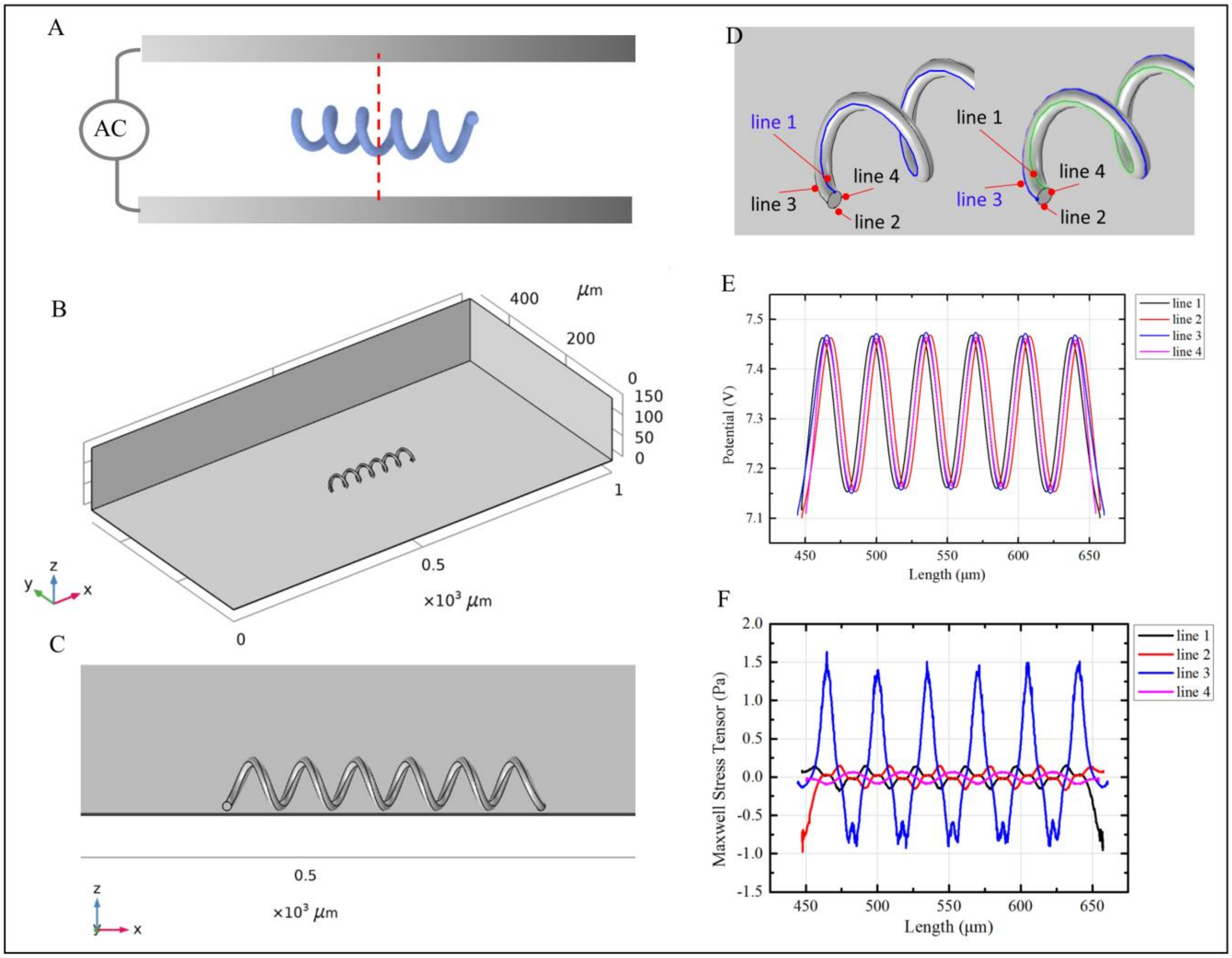

Supplementary Figure 3. (A) A schematic of the simulation model. (B)-(C) Simulation model. (D) The four contour lines on the surface of micro-spiral for the electric character. Simulation results of (E) electric potential and (F) Maxwell stress tensor of the four contour lines.

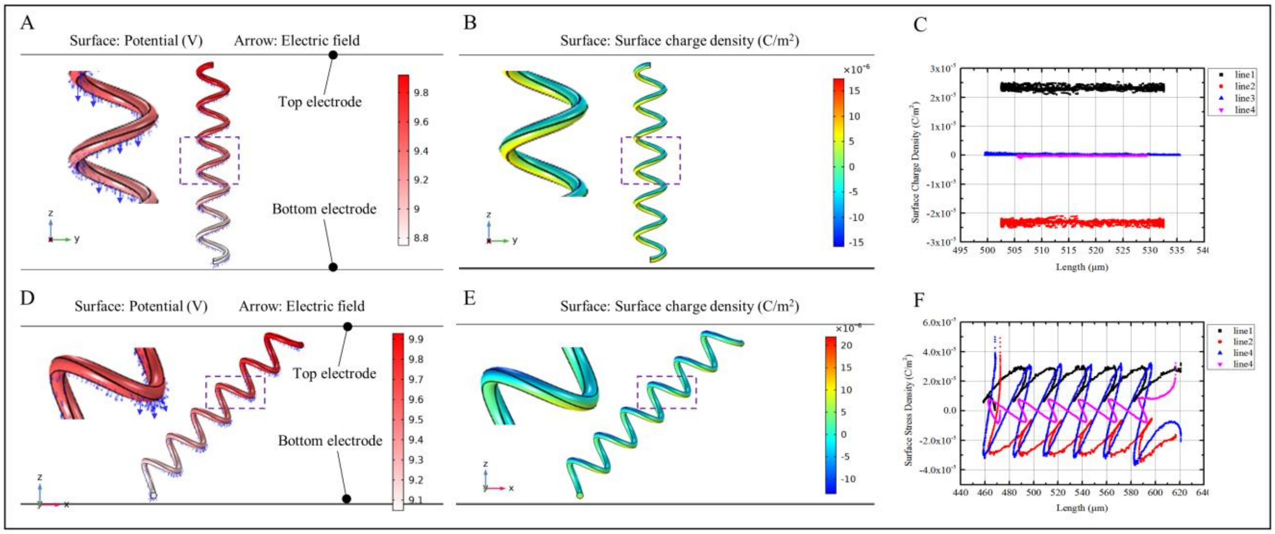

Supplementary Figure 4. Simulation results of two differently orientated spirals. Electric potential (A) and surface charge density (B) and (C) distribution of the model with the angle of 90°. Electric potential (D) and surface charge density (E) and (F) distribution of the model with the angle of 45°.

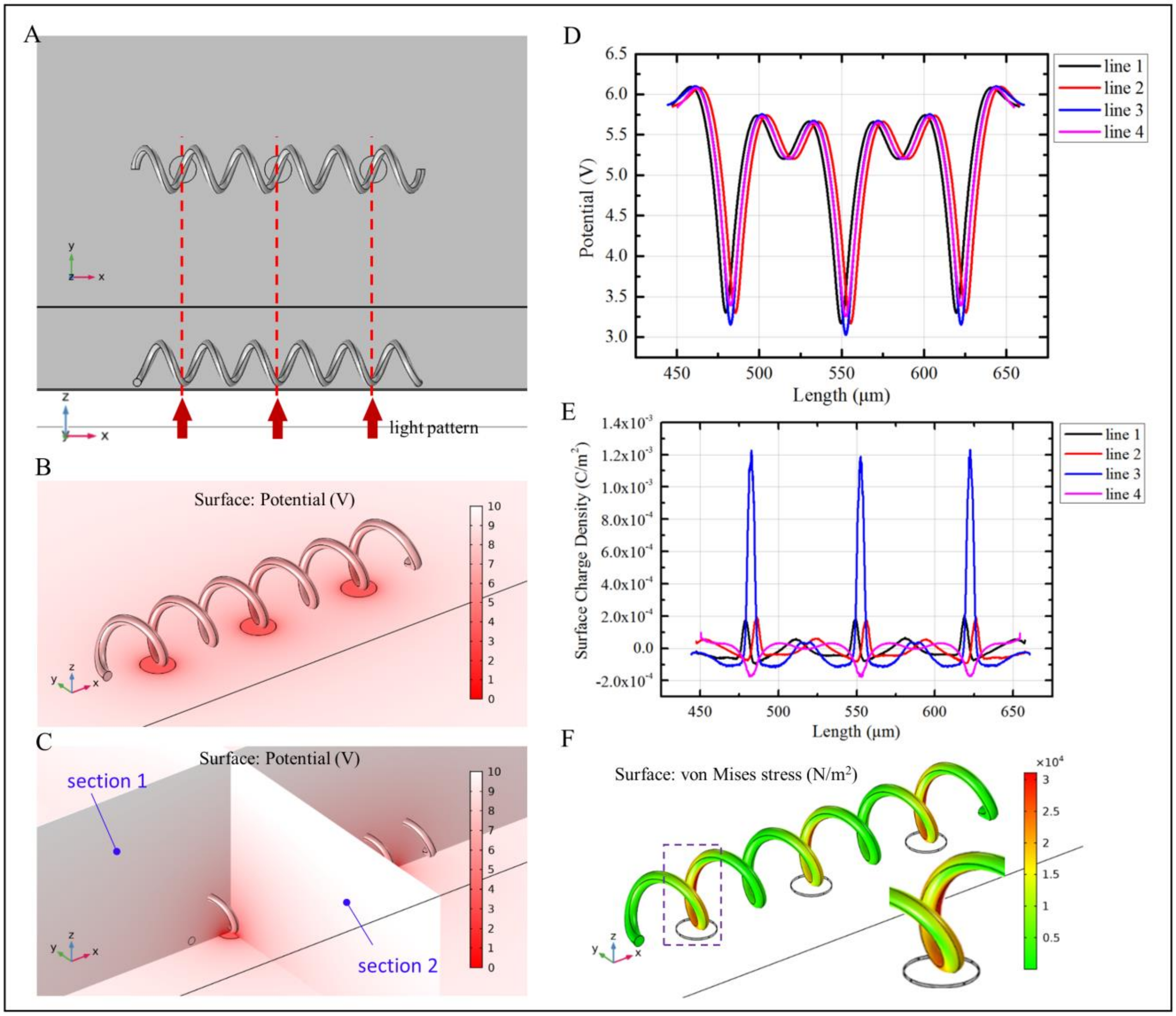

Supplementary Figure 5. (A)Simulation model for multiple light spots manipulation. Simulation results of (B)-(D) the electric potential, (E) surface charge density, and (F) stress tensor of micro-spiral under multiple light spots illumined in the OET system.

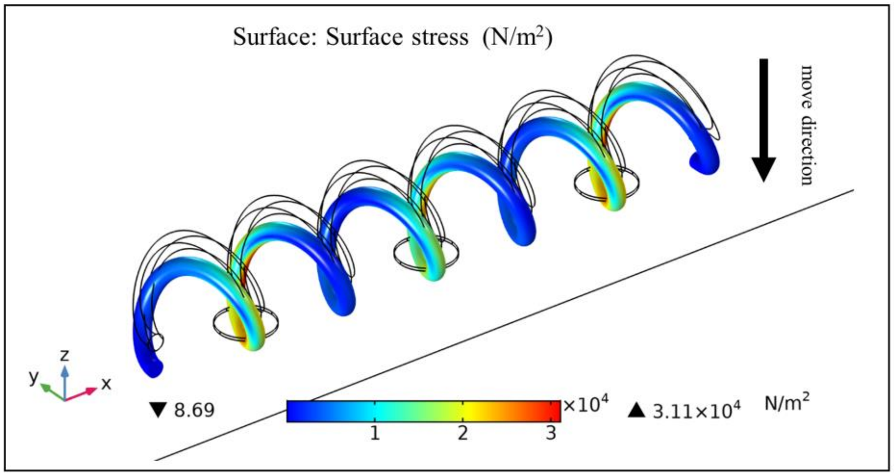

Supplementary Figure 6. The simulation results of surface stress and moving of micro-spiral.

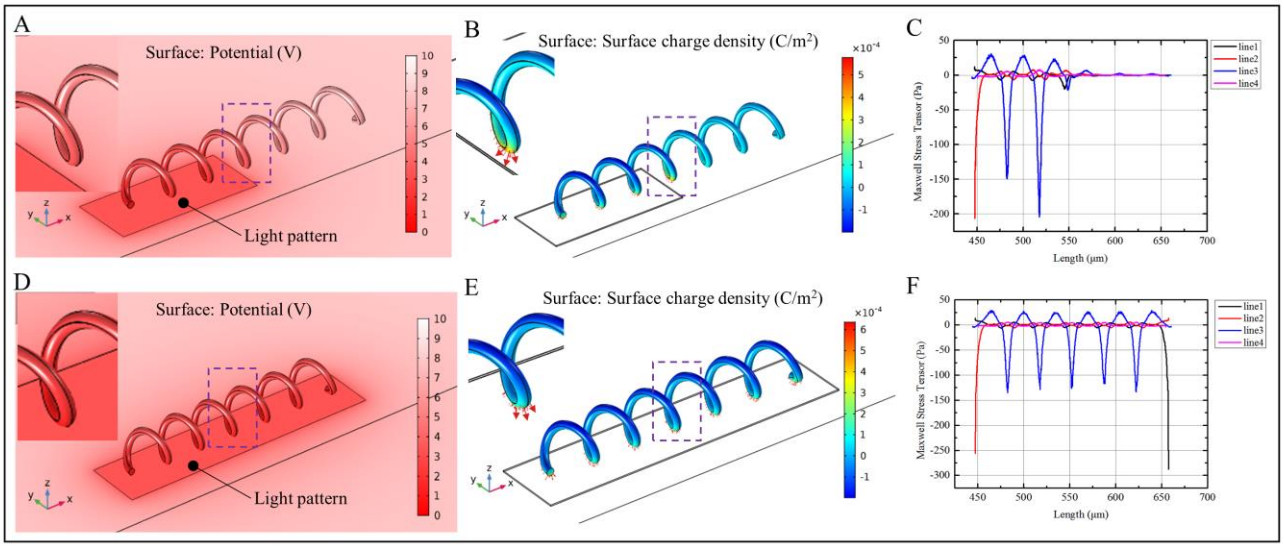

Supplementary Figure 7. Simulation results of two differently light patterns. Electric potential (A), surface charge density (B), and Maxwell stress tensor (C) distribution of the model with a little bit bigger than a half of the length of micro-spiral. Electric potential (D), surface charge density (E), and Maxwell stress tensor (F) distribution of the model with a little bit bigger than the length of micro-spiral.

3 Supplementary Videos

Video clip 1: The MST simulation results and cooperatively manipulation of multiple light spots;

Video clip 2: Parallel translated the micro-spirulina by multiple light spots in the OET system in the same direction and opposite direction;

Video clip 3: Parallel rotated the micro-spirulina by multiple light spots in the OET system along with a point in the bottom electrode and a point of itself.

Video clip 4: Flexible assemble micro-spiral into the different shapes of micro-structure.

4 Supplementary Reference

Jubery T. Z., Srivastava S. K. and Dutta P. (2014). Dielectrophoretic separation of bioparticles in microdevices: A review *Electrophoresis* 35: 691-713. doi:10.1002/elps.201300424.

Gagnon Z. R. (2011). Cellular dielectrophoresis: Applications to the characterization, manipulation, separation and patterning of cells *ELECTROPHORESIS* 32: 2466-2487. doi:10.1002/elps.201100060.

Qian C., Huang H., Chen L., Li X., Ge Z., Chen T., Yang Z. and Sun L. (2014). Dielectrophoresis for bioparticle manipulation *Int J Mol Sci* 15. doi:10.3390/ijms151018281.

Li X., Cai J., Sun L., Yue Y. and Zhang D. (2016). Manipulation and assembly behavior of spirulina-templated microcoils in the electric field *Rsc Advances* 6: 76716-76723. doi:10.1039/C6RA06344F.

Tao Q., Lan F., Jiang M., Wei F. and Li G. (2015). Simulation study of dielectrophoretic assembly of nanowire between electrode pairs *Journal of Nanoparticle Research* 17: 306. doi:10.1007/s11051-015-3102-6.

Dalir H., Yanagida Y. and Hatsuzawa T. (2009). Probing DNA mechanical characteristics by dielectrophoresis *Sensors and Actuators B: Chemical* 136: 472-478. doi:10.1016/j.snb.2008.11.004.

Zhang S., Erica Y. S., Jastaranpreet S., Yujie C. and Aaron R. W. (2019). The optoelectronic microrobot: A versatile toolbox for micromanipulation *Proceedings of the National Academy of sciences* 116: 14823-14828. doi:10.1073/pnas.1903406116.

Liang W., Liu L., Wang J., Yang X. and Yang W. (2020). A review on optoelectrokinetics-based manipulation and fabrication of micro/nanomaterials *Micromachines* 11: 78. doi:10.3390/mi11010078.
